# p53 expression in pterygium in two climatic regions in Turkey

**DOI:** 10.4103/0301-4738.49394

**Published:** 2009

**Authors:** Aysel Pelit, Nebil Bal, Yonca A Akova, Beyhan Demirhan

**Affiliations:** Department of Ophthalmology, Başkent University School of Medicine, Adana Teaching and Medical Research Center, Adana, Turkey; 1 Department of Pathology, Başkent University School of Medicine, Adana Teaching and Medical Research Center, Adana, Turkey

**Keywords:** Conjunctiva, pterygium, tumor supressor gene p53

## Abstract

**Purpose:**

To assess accumulation of p53 protein in samples of primary pterygium from people living in two different climatic regions in Turkey.

**Materials and Methods:**

Group 1 included 101 pterygium specimens from people in Adana located in southern Turkey. Group 2 included 39 pterygium specimens from people in Ankara, located in the middle of Turkey. Climatic conditions throughout the year are sunnier and warmer in Adana than they are in Ankara. The control group (Group 3) included 30 specimens of conjunctiva that had been excised during cataract surgery from 30 patients without pterygium. The pterygial specimens and control conjunctiva were studied by immunohistochemistry using antibodies against p53 protein. Pearson's chi-square test was used to compare the p53 immunoreactivity.

**Results:**

The p53 immunoreactivity in Groups 1 and 2 was greater than it was in the control group (*P<*0.001). There were no differences in p53 immunoreactivity between Groups 1 and 2 (*P=* 0.060).

**Conclusion:**

The p53 immunoreactivity was not correlated with ultraviolet irradiation exposure. The p53 immunoreactivity in our pterygium specimens suggests that pterygium could be a result of uncontrolled cell proliferation.

Pterygium is a common, benign, fibrovascular lesion, originating from the bulbar conjunctiva or the basal limbal epithelium. The pathogenesis of pterygium is not well understood. The most widely recognized etiologic factor is ultraviolet irradiation.[1] Until recently, pterygium was considered a degenerative disorder involving mainly the subepithelial tissue. However, more recently, tumor-like characteristics have been found in pterygia. Recent reports suggest that pterygium should be considered a benign neoplastic lesion.[2,3]

Molecular genetic studies have demonstrated that in human neoplastic tissues *in vivo*, excess p53 protein accumulation in cells, detectable by immunohistochemistry, is synonymous with mutations in the p53 gene.[4–6] Mutations in the p53 gene are the most common genetic marker of human neoplastic growth. The human p53 gene is located on the short arm (p) of chromosome 17 and encodes for a 393-amino acid, nuclear phosphoprotein cell cycle transcription factor.[7] The p53 gene product is involved in the inhibition of cell proliferation by preventing cells from entering S-phase[8–11] and allows cells with damaged DNA to be eliminated by the p53-dependent programmed cell death mechanism.[12] In normal cells, wild-type p53 proteins have a short half-life and do not accumulate in large amounts. Thus, they are almost undetectable by immune tests, including immunohistochemistry.[13,14] Mutations in the p53 gene can lead to the synthesis of abnormal p53 proteins, which have altered conformations with loss of DNA binding[15] and complex to cellular proteins, resulting in prolonged half-lives and accumulation in the cells.[8] Accumulated mutant p53 proteins, which are often implicated as one of main steps in transforming altered cells into tumor cells, can then be detected, usually by immunohistochemistry, in neoplastic tissues.[8,16]

The aim of the study was to investigate the accumulation of p53 protein in the pterygium specimens from persons living in two different climatic regions in Turkey.

## Materials and Methods

Informed consent, according to the tenets of the Declaration of Helsinki, was obtained before examination. The study was approved by the local ethics committee [KA03/52]. We divided the study samples into the two groups. Group 1 (n = 101) included samples from persons in Adana, located in southern Turkey. Group 2 (n = 39) included samples from persons in Ankara located in the middle of Turkey. Recurrent pterygia were not included in the study. Group 3 was the control group (n = 30) and these patients were residents of Adana.

Climatic conditions are sunnier and warmer throughout the year in Adana than they are in Ankara. Patients from both Adana and Ankara worked outdoors. None of the patients wore sunglasses.

The place of birth was Adana and the surrounding district in Group 1. The place of birth was Ankara and the surrounding district in Group 2. All cases had lived in their region for their whole life.

We asked the meteorology institute about ultraviolet (UV) radiation level in Adana and Ankara. They don't have UV radiation results for Adana. They gave us Antalya UV radiation results for 2001. Antalya is located as far south as Adana. Antalya has the same climatic conditions as Adana. Ankara is located in the middle of Turkey.

There were statistically significant differences regarding the UV radiation for Antalya and Ankara (*P* < 0.001). UV radiation in Antalya was significantly higher than UV radiation in Ankara. Mean UV radiation during 2001 for Antalya was 0.74 ± 0.64 Med/hr, and mean UV radiation during 2001 for Ankara was 0.63 ± 0.55Med/hr.

The control group consisted of samples of normal conjunctival tissue that had been excised during cataract surgery from 30patients without pterygium. The control tissue was excised at the same site as a pterygium would have been located.

All the pterygium and control conjunctiva specimens were fixed in 10% formalin solution for 24h. The specimens were mounted in paraffin blocks. Five-µm thick sections were cut and then deparaffinized and briefly washed in alcohol, followed by 2–5 min of washing in phosphate buffer solution (PBS, pH 7.4).

Endogenous peroxidase activity was blocked by immersion for 30 min in 3% H_2_O_2_ in methanol at room temperature, followed by 3–5 min washing in distilled water. Sections then were immersed in 10 mm citrate buffer (pH 6.0) and heated in a microwave oven for 50 min to increase expression of the antigen. After removing the container from the microwave oven and cooling it for 50 min, slides were placed in phosphate buffer saline (PBS) (pH 7.4) for 2 to 3 min. Sections were then treated with bovine serum albumin to prevent background staining and incubated for 2 h with monoclonal mouse anti-human mutant p53 protein (Clone:DO-7 Dako, Copenhagen, Denmark). After washing in PBS (Lab Vision Corp/Neomarkers, Fremont, Calif, USA), biotinylated goat anti-mouse (Lab Vision, TM-060-HL) antibody was applied for 20 min at room temperature, and sections were washed for 3 min in PBS. This was followed by incubation with streptavidin-biotinylated peroxidase (Lab Vision, TM-060-HL) complex for 30 min, and sections were washed for 3 min in PBS. Sections were developed in 3-amino-9-ethylcarbazole (AEC) (Dako, Copenhagen, Denmark) for 5–10min with microscopic control. Slides were lightly counterstained with Mayer's hematoxylin, dehydrated, and mounted in the usual manner.

Since AEC was used as the color reagent, bright red or orange-brown was considered as a positive indication of p53 binding in squamous epithelium of the specimens. Positive staining was evaluated as nuclear staining for p53. Negative staining was defined as when less than 5% of the epithelial cells showed distinct nuclear staining for p53.

The Student *t* test was used for age, the size of pterygium, and UV radiation level comparison. Pearson's chi-square test was used to compare the p53 immunoreactivity (SPSS ver.8.0).

Values for *P* less than 0.05 were considered statistically significant.

## Results

There were no statistically significant differences regarding the mean ages of patients in Groups 1 and 2 and in the control group (56.71 ± 13.69 years, range 42–78 years; 55.85 ± 13.59 years, range 40–72 years; and 56.80 ± 12.99 years, range 44–69 years).

The study included identical samples from both groups. There were no statistically significant differences regarding the size of the pterygium (extent of overgrowth into cornea) in Groups 1 and 2 (*P = 0.868*) (3,58 ± 0.67 mm, 3,56 ± 0.68mm, respectively).

**Figure 1:**
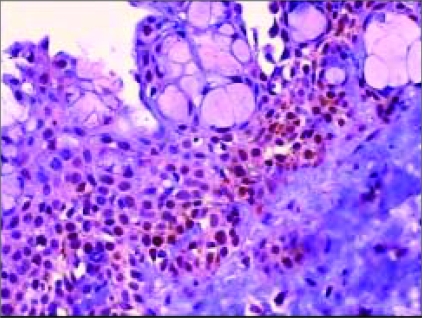
Immunohistochemical staining with monoclonal antibody DO-7 on section of pterygium showing positive staining (brown nuclei) for p53, mainly in the basal layer of the epithelium (×200)

Incidence of positive staining for mutant p53 was 60.4% in Group 1, 74.5% in Group 2, and 13.3% in the control group Table 1, [Fig. 1]. Differences in p53 immunoreactivity between Groups 1 and 2 versus the control group were significant (*P < 0.001*). p53 immunoreactivity was greater in Groups 1 and 2 than it was in the control group (*P < 0.001*). Differences in p53 immunoreactivity between Groups 1 and 2 were not significantly different (*P = 0.060*).

**Table 1:** Positivity for mutant p53 in group 1, 2 and control group

	Mutant P53 (−)	Mutant P53 (+)	Total
Group 1	40 (39.6)	61 (60.4)	101
Group 2	10 (25.5)	29 (74.5)	39
Group 3 (Control)	26 (86.7)	4 (13.3)	30

Figures in parentheses are in percentage

## Discussion

In our study, we examined expression of the p53 tumor suppressor gene in patients from the Turkish cities of Adana and Ankara, which have different climatic conditions. The southern city of Adana and its surrounding area has very sunny conditions between April and October, so the population there experiences high levels of UV radiation exposure. In contrast, the more central city of Ankara receives much less sunshine and people in that area receive significantly less UV radiations. We found that p53 immunoreactivity was significantly greater in the pterygium groups than it was in the control group. However, we found no difference in p53 expression when samples from people in two different climatic regions were compared.

The geographical variations in the incidence of diseases such as pterygium and droplet keratopathies have led to theories pointing to sunlight and UV exposure as potential etiologic factors. Epidemiological studies, indicate that chronic exposure to the sunlight, and most probably UV radiation, is an important factor in the development of pterygia, but the mechanism by which UV radiation induces this disease remains unknown.[17,18] It is known that UV radiation has a carcinogenic effect resulting in DNA damage with loss of normal growth control. In normal cells, the p53 protein has a short half-life and is maintained at low, often undetectable levels. Mutation in the p53 gene is believed to lead to an increased stability of the protein, allowing its more pronounced immunohistochemical detection. UV radiation can cause mutation in genes such as p53, which when inactivated through mutation and loss of heterozygosity can lead to cell proliferation and genomic instability.[2] In our report, UV radiation in Adana was significantly higher than UV radiation in Ankara. We observed that there was no significant difference between Adana and Ankara pterygium patients in p53 immunoreactivity (*P = 0.060*). We feel that a certain level of UV exposure might have caused a failure in the control of the cell cycle in limbal epithelial cells in the samples from pterygium patients from Adana and Ankara regions.

Pterygia can be described as hidden limbal tumors which arise from the limbal epithelium. Initially, pterygia are clinically invisible while growing concentrically in the interpalpebral limbal region, because their cell layers are suppressed in numbers. Dushku *et al*.,[19] showed that pterygia are caused by a mutation in limbal epithelial basal cells. While in the limbal region, pterygia infiltrate centrifugally into the adjacent conjunctival epithelium, the circumferential limbal epithelium, and the corneal epithelium.

Weinstein *et al.*[20] found no difference in p53 expression between primary pterygia samples and those of recurrent pterygia. They suggest that abnormal p53 expression might imply that the samples contained transformed cells and that there is a failure in the regulation and control of the cell cycle. In that study, the authors concluded that pterygium is a growth disorder rather than degeneration.

Pterygium formation has been reported to be related to dose of UV irradiation.[21] UV irradiation mainly produces DNA lesions between adjacent pyrimidines, and C to T transitions on dipyrimidine sites or CC to TT tandem mutations in the p53 gene are considered as the UV-related skin cancer molecular signatures.[22] Tsai *et al*.,[6] found that there was one case with a C to T transition, but no CC to TT tandem mutations in their 51 patients undergoing pterygium surgery. They suggested that besides p53 gene mutations, there may be other mechanisms leading to loss of p53 function involved.

In the current study, incidence of negative staining for mutant p53 gene was 39.6% in Group 1 and 25.5% in Group 2. Incidence of positive staining for mutant p53 gene was 13.3% in the control group. This indicates that there are other mechanisms not yet known which may also independently lead to pterygium formation.

In conclusion, the expression of p53 might be the result of UV exposure at a certain level which could be enough to trigger the mitotic reaction in both populations, with no relation to the whole annual amount of radiation. In such a case the difference between the climate in Adana and Ankara has no impact on the presented finding. We do know that UV damage has a quantitative accumulation property. Outdoor work in both climates may provide the required radiation to start the pathologic process.
